# Prognostic impact of high-sensitive troponin on 30-day mortality in patients with acute heart failure and different classes of left ventricular ejection fraction

**DOI:** 10.1007/s00380-022-02026-x

**Published:** 2022-01-15

**Authors:** Jakob Ledwoch, Jana Kraxenberger, Anna Krauth, Alisa Schneider, Katharina Leidgschwendner, Vera Schneider, Alexander Müller, Karl-Ludwig Laugwitz, Christian Kupatt, Eimo Martens

**Affiliations:** 1 Klinik und Poliklinik für Innere Medizin I, Klinikum rechts der Isar, Technical University of Munich, Munich, Germany; 2 Klinik für Kardiologie, Pneumologie und Internistische Intensivmedizin, München Klinik Neuperlach, Munich, Germany; 3 DZHK (German Center for Cardiovascular Research), Partner Site Munich Heart Alliance, Munich, Germany

**Keywords:** Outcome prediction, Acute heart failure, Troponin, HFpEF, HFrEF, HFmrEF

## Abstract

High-sensitive troponin T (hs-TnT) is increasingly used for prognostication in patients with acute heart failure (AHF). However, uncertainty exists whether hs-TnT shows comparable prognostic performance in patients with heart failure and different classes of left ventricular ejection fraction (LV-EF). The aim of the present study was to assess the prognostic value of hs-TnT for the prediction of 30-day mortality depending on the presence of HF with preserved ejection fraction (HFpEF), HF with mid-range LV-EF (HFmrEF) and HF with reduced LV-EF (HFrEF) in patients with acutely decompensated HF. Patients admitted to our institution due to AHF were retrospectively included. Clinical information was gathered from electronic and paper-based patient charts. Patients with myocardial infarction were excluded. A total of 847 patients were enrolled into the present study. A significant association was found between HF groups and hs-TnT (regression coefficient -0.018 for HFpEF vs. HFmrEF/HFrEF; *p* = 0.02). The area under the curve (AUC) of hs-TnT for the prediction of 30-mortality was significantly lower in patients with HFpEF (AUC 0.61) than those with HFmrEF (AUC 0.80; *p* = 0.01) and HFrEF (AUC 0.73; *p* = 0.04). Hs-TnT was not independently associated with 30-day outcome in the HFpEF group (OR 1.48 [95%-CI 0.89–2.46]; *p* = 0.13) in contrast to the HFmrEF group (OR 4.53 [95%-CI 1.85–11.1]; *p* < 0.001) and HFrEF group (OR 2.58 [95%-CI 1.57–4.23]; *p* < 0.001). Prognostic accuracy of hs-TnT in patients hospitalized for AHF regarding 30-day mortality is significantly lower in patients with HFpEF compared to those with HFmrEF and HFrEF.

**Supplementary Information:**

The online version contains supplementary material available at 10.1007/s00380-022-02026-x.

## Introduction

There is increasing evidence regarding the prognostic potential of cardiac troponin in patients with acute heart failure (AHF) [1–4]. The prognostic accuracy of troponin in this setting was further improved after the introduction of high-sensitive assays compared to conventional measurements [5]. Particularly, prediction of short-term outcome can help to identify AHF patients at increased risk during the initial period after decompensation. Such patients might benefit from more aggressive HF treatment and/or a closer follow-up program. Furthermore, troponin is increasingly used for triage of patients with AHF in the emergency department [6, 7]. In this setting, precise prognostication is crucial when using this parameter. However, the majority of evidence regarding the prognostic role of troponin in AHF comes from studies analyzing HF with reduced EF (HFrEF) [1, 4, 7, 8] and only little is known about its use in HF with preserved ejection fraction (HFpEF) compared to HF and mid-range EF (HFmrEF) as well as to HF and reduced EF (HFrEF). The few available studies analyzing troponin in these different HF groups including HFpEF and HFmrEF were limited by inclusion of only stable HF patients [9], assessing only long-term outcome [10] or using conventional instead of high sensitivity troponin assays [3, 11]. This represents a relevant diagnostic dilemma since approximately 40–50% of patients admitted for AHF have HFpEF [12, 13]. Although there are well-validated risk scores in AHF such as the MEESSI score [14, 15], data regarding adequate prognostication using biomarkers in these patients are scarce. Therefore, the present study sought to evaluate hs-TnT based clinical outcome prediction in patients with different HF groups (HFpEF, HFmrEF and HFrEF) in a large all-comer AHF population.

## Materials and methods

### Study cohort

Patients aged ≥ 18 years presenting with AHF in our institution were included into a single-center retrospective AHF registry. Participants were enrolled between 2012, the year when high-sensitive troponin T (hs-TnT) was implemented into clinical routine in our institution, and 2019. Patient informed consent was waived due to the retrospective nature of the study. The study was approved by the hospital’s ethics committee and performed according to the Decleration of Helsinki.

AHF was diagnosed according to current guidelines [16]. It included new onset of HF and acute decompensation of chronic HF. For the present analysis, patients were categorized into three different groups of HF and LV-EF according to current guidelines (HFpEF vs. HFmrEF vs. HFrEF) [16]. Exclusion criteria used were missing hs-TnT, no or incomplete echocardiography, patients with respiratory failure (defined as need for mechanical ventilation), cardiogenic shock (defined as systolic blood pressure < 90 mm Hg or need for catecholamine therapy to maintain a systolic pressure ≥ 90 mm Hg together with clinical signs of impaired end-organ perfusion) and myocardial infarction, which was diagnosed according to the fourth universal definition of myocardial infarction [17]: Rise and/or fall of hs-TnT with at least one value above the 99th percentile URL and with at least one of the following: (I) symptoms suggestive for acute coronary syndrome (chest pain or dyspnea); (II) new ischemic ECG changes (ST-segment depression, T-wave inversion, new pathological Q waves); (III) new wall motion abnormalities consistent with ischemic aetiology and (IV) identification of coronary thrombus by invasive angiography. Patients with rise and/or fall of hs-TnT with at least one value above the 99th percentile URL and clinical symptoms (chest pain or dyspnea) but without other criteria for myocardial infarction (new ischemic ECG changes, new wall motion abnormalities consistent with ischemic etiology) were classified as not having myocardial infarction [17].

### Patient data assessment

Patient data were extracted from electronic charts and paper-based document files. In each patient information regarding medical history, clinical signs and symptoms on hospital admission, ECG results, echocardiographic examinations and laboratory measurements were obtained. LV-EF assessment was performed by modified biplane Simpson´s method according to current guidelines [18]. Clinical outcome was assessed until 30 days following hospital admission.

### Laboratory measurements and endpoint definition

Concentration of hs-TnT was analyzed by Elecsys hs-TnT assay (Roche Diagnostics, Basel, Switzerland). At least two hs-TnT measurements were performed in each patient within 6 h following hospital admission. Renal function was quantified by an estimated glomerular filtration rate (eGFR) using the Chronic Kidney Disease Epidemiology Collaboration formula [19].

Primary outcome measure was the assessment of the predictive value of maximum hs-TnT within 6 h of hospital admission with respect to 30-day mortality in different classes of HF.

### Statistical analysis

Continuous variables were tested for normal distribution by Kolmogorov–Smirnov using Lilliefors correction and reported as median with interquartile range (IQR) or means with standard deviation. Categorical variables were expressed as numbers and percentages. Between-group comparisons across the three different HF groups (HFpEF vs. HFmrEF vs. HFrEF) were performed using Kruskal–Wallis test or ANOVA for continuous data and Chi-Square or Fisher´s exact test for categorial data. A multivariable linear regression was conducted to identify factors independently associated with hs-TnT. Variables were included into this model in case of significance in the univariate analysis. Furthermore, receiver operating characteristic (ROC) analyses were performed to assess the area under the curve (AUC) for maximum hs-TnT within 6 h of admission in predicting 30-day mortality in different groups of LV-EF. AUC comparison between these groups was performed using the z-test. The mortality prediction analysis included the calculation of a ROC derived hs-TnT cut-off using the Youden index defined by the minimal distance of the ROC curve to the point (0;1) of the graph. In order to assess hs-TnT as independent predictor for 30-day mortality in the three different HF groups multivariable logistic regression was performed adjusted for age, sex, NYHA class, diabetes mellitus, arterial hypertension, myocardial infarction, atrial fibrillation and eGFR. Hs-TnT was log-transformed (natural logarithm [ln]) for this model to adjust for the exponential distribution of its values. Hypothesis testing was two-tailed and a *p*-value < 0.05 was considered as significant. Statistical analyses were performed using SPSS, version 26 (IBM, Chicago, USA).

## Results

### Study population and baseline characteristics

**Fig. 1:**
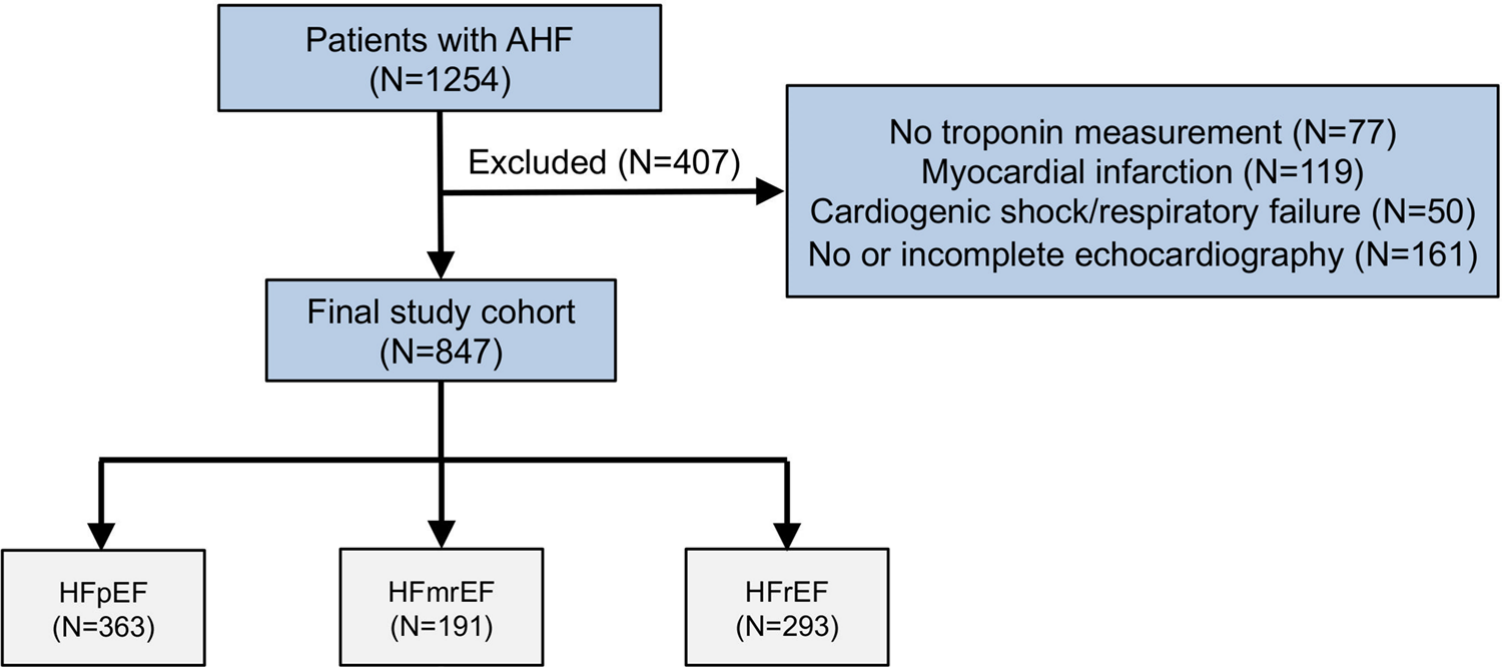
Study flow chart. *AHF* Acute heart failure, *HFpEF* Heart failure with preserved ejection fraction, *HFmrEF* Heart failure with mid-range reduced ejection fraction, *HFrEF* Heart failure with reduced ejection fraction

After exclusion of 407 patients from the initial cohort of individuals admitted due to AHF, 847 patients were available for the current analysis. Of them, 363 patients (43%) had HFpEF, 304 patients (23%) had HFmrEF and 293 patients had HFrEF (35%). The study flow is illustrated in Fig. 1.

Baseline characteristics across the different HF groups are presented in Table 1. Patients with HFpEF were older and were more frequently female. Cardiovascular ischemic factors such as coronary artery disease, history of myocardial infarction and previous revascularization procedures were less often in HFpEF patients. With respect to initial clinical presentation of the patients no differences were observed except for NYHA grade ≥ III being less often present in the HFpEF group. Regarding echocardiographic measures, LV diameter were smaller and RV systolic function was higher in HFpEF patients compared to those with HFmrEF and HFrEF.

**Table 1:** Baseline characteristics

	HFpEF (*N* = 363)	HFmrEF (*N* = 191)	HFrEF (*N* = 293)	*p*-value
Age (years)	80 (73–85)	80 (74–86)	76 (67–83)	< 0.001
Female	57% (208)	39% (74)	32% (94)	< 0.001
Arterial hypertension	88% (320)	92% (175)	78% (229)	< 0.001
Dyslipidemia	41% (149)	51% (98)	43% (126)	0.06
Smoker	32% (116)	32% (61)	38% (111)	0.22
Diabetes mellitus	31% (114)	42% (80)	33% (98)	0.04
Coronary artery disease	41% (149)	62% (119)	52% (152)	< 0.001
Previous MI	11% (41)	26% (50)	26% (78)	< 0.001
Previous PCI	23% (82)	35% (66)	32% (93)	0.004
Previous CABG	6% (21)	17% (32)	12% (36)	< 0.001
Atrial fibrillation	66% (239)	74% (141)	58% (169)	0.001
Clinical signs and symptoms				
Pulmonary congestion	75% (272)	83% (159)	78% (229)	0.08
Peripheral edema	76% (275)	75% (143)	71% (209)	0.42
NYHA ≥ III	90% (325)	96% (184)	95% (278)	0.003
Vital signs				
Oxygen saturation (%)	93 (88–97)	93 (88–96)	94 (88–97)	0.27
Systolic blood pressure (mmHg)	143 (125–160)	143 (126–160)	130 (114–150)	< 0.001
Heart rate (beats/min)	80 (67–98)	84 (68–108)	90 (75–107)	< 0.001
Echocardiographic results				
LV-EF (%)	60 (50–60)	40 (40–45)	30 (26–33)	< 0.001
LVEDD (mm)	47 (42–52)	50 (45–57)	56 (50–63)	< 0.001
LVESD (mm)	32 (28–36)	38 (32–44)	46 (40–54)	< 0.001
TAPSE (mm)	18 (15–22)	17 (14–20)	15 (12–18)	< 0.001
Laboratory findings				
eGFR (ml/min)	57 (35–78)	55 (37–77)	55 (39–76)	0.99
Haemoglobin (g/dl)	12.1 (10.3–13.6)	12.0 (10.3–13.6)	12.7 (11.1–14.5)	< 0.001
Maximum hs-TnT (ng/l) within 6 h of admission	28 (18–46)	39 (23–61)	40 (24–68)	< 0.001
Maximum hs-TnT > 99th percentile URL (14 ng/l)	82% (298)	90% (172)	92% (268)	0.001

*CABG* Coronary artery bypass graft, *eGFR* Estimated glomerular filtration rate, *HFpEF* Heart failure with preserved ejection fraction, *HFmrEF* Heart failure with mid-range reduced ejection fraction, *HFrEF* Heart failure with reduced ejection fraction, *Hs-TnT* High-sensitive troponin T, *LV-EF* Left ventricular ejection fraction, *LVEDD* Left ventricular enddiastolic diameter, *LVESD* Left ventricular endsystolic diameter, *MI* Myocardial infarction, *PCI* Percutaneous coronary intervention, *TAPSE* Tricuspid annular plane systolic excursion, *URL* upper reference limit

### Association of HF groups with hs-TnT levels

Laboratory findings in patients with HFpEF, HFmrEF and HFrEF are listed in Table 1. Hs-TnT was significantly higher in patients with HFmrEF and HFrEF (HFmrEF vs. HFpEF *p* < 0.001; HFrEFvs. HFpEF *p* < 0.001). Increased hs-TnT above the 99th percentile URL (> 14 ng/l) was found in 82% of patients with HFpEF, 90% of patients with HFmrEF and 92% with HFrEF (*p* = 0.001). In the multivariable linear regression model HF groups were independently associated with hs-TnT (HFpEF versus HFmrEF/HFrEF was significantly associated with lower hs-TnT) (Table 2). A linear regression depicting factors independently associated with hs-TnT in the three respective HF groups are outlined in Supplemental Table S1.

**Table 2:** Independent association with maximum hs-TnT

	Regression coefficient ß	*p*-value
Age	0.001	0.97
Arterial hypertension	– 0.016	0.12
Dyslipidemia	– .007	0.38
Smoker	– 0.013	0.09
Diabetes mellitus	– 0.005	0.48
Coronary artery disease	0.013	0.09
Peripheral edema	– 0.019	0.02
Heart rate	0.001	0.47
eGFR	– 0.001	< 0.001
HFpEF vs. HFmrEF/HFrEF	– 0.018	0.02

*eGFR* Estimated glomerular filtration rate, *HFpEF* Heart failure with preserved ejection fraction, *HFmrEF* Heart failure with mid-range reduced ejection fraction, *HFrEF* Heart failure with reduced ejection fraction, *Hs-TnT* High-sensitive troponin T

### Prognostic value of hs-TnT regarding 30-day mortality in different HF groups

**Fig. 2:**
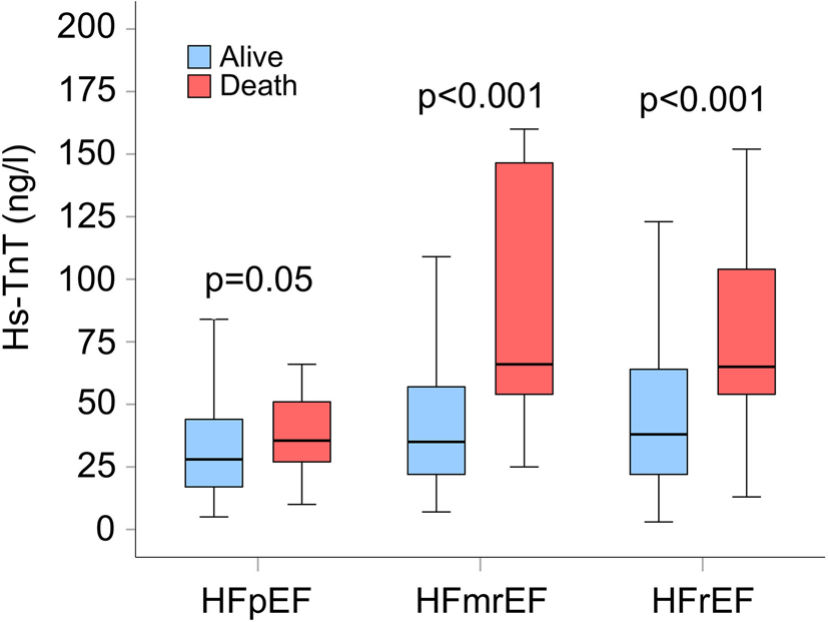
Hs-TnT levels depending on clinical outcome at 30 days in different HF groups. *Hs-TnT* High sensitive troponin T, *HFpEF* Heart failure with preserved ejection fraction, *HFmrEF* Heart failure with mid-range reduced ejection fraction, *HFrEF* Heart failure with reduced ejection fraction

In the present study 30-day mortality was 8.9% (*n* = 75) without a significant difference between the groups of HFpEF (8.3%; *N* = 30), HFmrEF (6.3%; *N* = 12) and HFrEF (11.3%; *N* = 33) (*p* = 0.15). Hs-TnT was significantly higher among patients who died within 30-days after hospital admission compared to those alive in the group with HFmrEF and in the group with HFrEF (Fig. 2). In the group with HFpEF, there was a trend towards higher hs-TnT in patients who died compared to those alive, however without reaching statistical significance (*p* = 0.05) (Fig. 2).

**Fig. 3:**
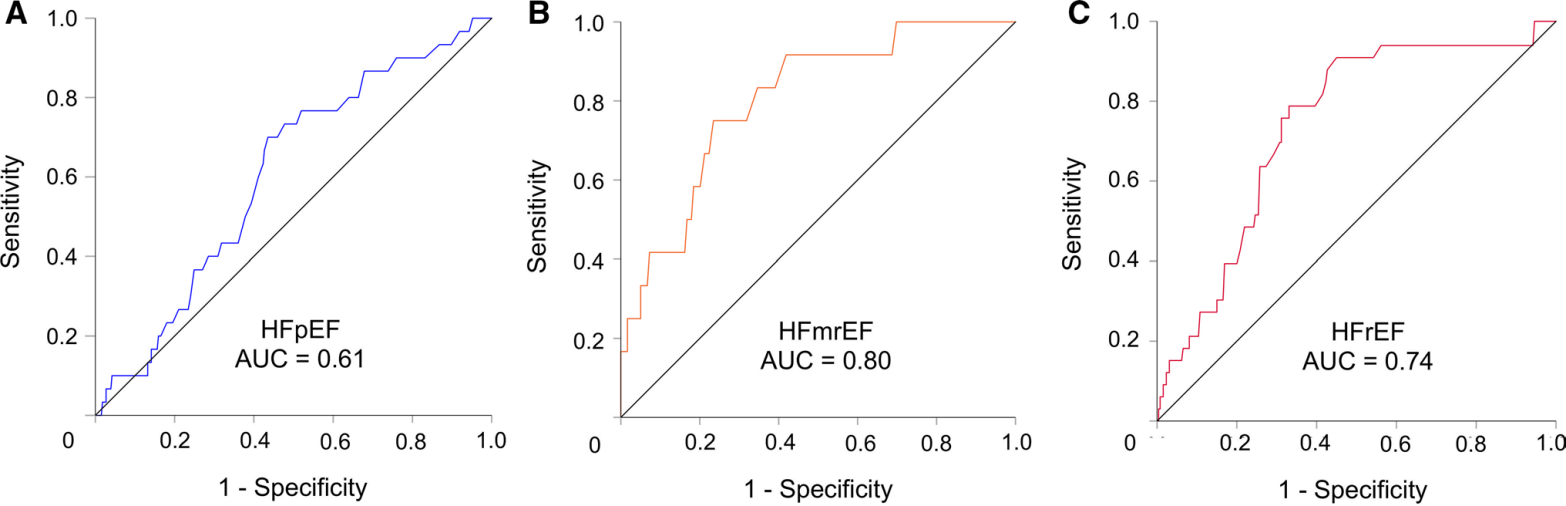
Area under the curve for hs-TnT regarding the prediction of 30-day mortality in patients with HFpEF (panel A), HFmrEF (panel B) and HFrEF (panel C). *AUC* Area under the curve, *HFpEF* Heart failure with preserved ejection fraction, *HFmrEF* Heart failure with mid-range reduced ejection fraction, *HFrEF* Heart failure with reduced ejection fraction

A ROC analysis of hs-TnT predicting 30-day mortality in all three HF groups is displayed in Fig. 3. The AUC of hs-TnT in HFpEF (AUC 0.61) was significantly lower compared to that in HFmrEF (AUC 0.80; AUC difference 0.21; *p* = 0.01) and in HFrEF (AUC 0.74; AUC difference 0.14; *p* = 0.04). No statistical difference regarding AUC was found between HFmrEF and HFrEF (AUC difference 0.06; *p* = 0.36). Table 3 depicts sensitivity and specificity of Youden Index optimized hs-TnT cut-offs and the 99th percentile upper reference limit (URL) hs-TnT cut-offs in the respective HF groups. The Youden Index optimized cut-offs provided markedly improved prediction of 30-day mortality compared to the 99th percentile URL, mainly by increasing specificity. The optimized hs-TnT cut-off in HFpEF patients was lower and showed inferior specificity compared to the optimized cut-off in HFmrEF and HFrEF patients.

**Table 3:** Youden Index optimized and 99^th^ percentile hs-TnT cut-offs regarding the prediction of 30-day mortality in different HF groups

	hs-TnT cut-off	Sensitivity	Specificity
HFpEF	29 ng/l (optimized)	73%	52%
	14 ng/l (99th percentile URL)	93%	13%
HFmrEF	60 ng/l (optimized)	75%	77%
	14 ng/l (99th percentile URL)	100%	7%
HFrEF	54 ng/l (optimized)	76%	69%
	14 ng/l (99th percentile URL)	94%	6%

*Hs-TnT* High-sensitive troponin T, *HFpEF*:Heart failure with preserved ejection fraction, *HFmrEF* Heart failure with mid-range reduced ejection fraction, *HFrEF* Heart failure with reduced ejection fraction, *URL* upper reference limit

The adjusted regression analysis of logarithmic hs-TnT showed a significant association with 30-day mortality in patients with HFrEF (OR 2.58 [95%-CI 1.57–4.23]; *p* < 0.001) and HFmrEF (OR 4.53 [95%-CI 1.85–11.1]; *p* < 0.001) but not in patients with HFpEF (OR 1.48 [95%-CI 0.89–2.46]; *p* = 0.13) (Fig. 3).

## Discussion

The present study is the first assessing the prognostic value of hs-TnT regarding short-term outcome in different classes of HF in a large cohort of AHF patients. The main result is that prediction of 30-day mortality using hs-TnT is less accurate in patients with HFpEF compared to HFmrEF and HFrEF.

### Association of hs-TnT levels with different HF groups

It is well known that acute decompensation in HF can lead to an increase in circulating cardiac troponin. This can be caused by different mechanisms including wall stress, altered calcium handling, endogenous catecholamines, oxidative stress and cytokines leading to cytosolic mobilization of troponin, apoptosis and/or cell necrosis [20]. The prevalence of elevated hs-TnT above the 99^th^ percentile URL (> 14 ng/l) vary between 77 and 90% in AHF [6, 21, 22]. Detection of abnormal hs-TnT levels in the setting of AHF occurs frequently as described above and indicate that acute decompensation induces almost always detectable troponin release above the 14 ng/l margin. This hs-TnT cut-off of 14 ng/l represents the 99th percentile URL of an apparently healthy population. In this setting, the 14 ng/l hs-TnT limit provides high discriminatory power to rule in /rule out myocardial infarction in patients presenting with chest pain [23]. Not surprisingly, the hs-TnT cut-off for 30-day mortality prediction in an AHF population is markedly higher compared to myocardial infarction diagnosis in apparently healthy patients.

With respect to different LV-EF classes, higher hs-TnT values are found among HFrEF and HFmrEF patients compared to those with HFpEF [10, 24]. This corresponds to the present work with a slightly higher proportion of elevated hs-TnT in HFmrEF and HFrEF compared to HFpEF. Furthermore, our adjusted analysis demonstrated HF classification based on LV-EF to be independently associated with hs-TnT (HFpEF associated with lower hs-TnT compared to HFmrEF/HFrEF). This result underlines our initial hypothesis of a strong association of hs-TnT with different classes of LV-EF in patients presenting with AHF. A possible explanation for this finding is the more frequently observed coronary artery disease in patients with HFmrEF and HFrEF compared to HFpEF. Coronary macroangiopathy or even microangiopathy can cause a mismatch of myocardial oxygen demand and supply during decompensation. This would lead to myocardial injury and contribute to hs-TnT release.

### Diagnostic value of troponin for the prediction of 30-day mortality

**Fig. 4:**
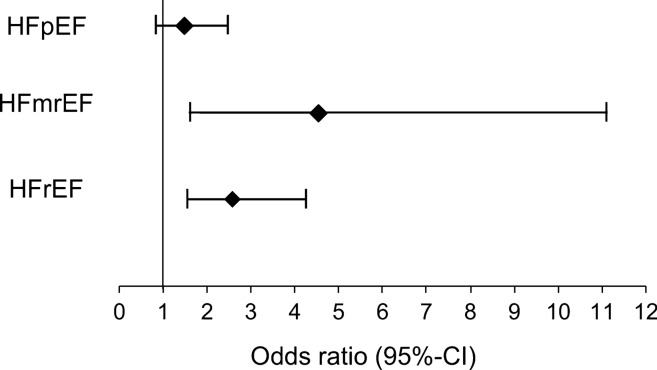
Multivariable adjusted analysis regarding the association of hs-TnT with 30-day mortality. Hs-TnT was log-transformed (natural logarithm) for this model to adjust for the exponential distribution of its values. Hs-TnT in this model was adjusted for age, sex, NYHA class, diabetes mellitus, arterial hypertension, myocardial infarction, atrial fibrillation and eGFR. *CI* Confidence interval, *HFpEF* Heart failure with preserved ejection fraction; *HFmrEF* Heart failure with mid-range reduced ejection fraction; *HFrEF* Heart failure with reduced ejection fraction

Since cardiac troponin is released from cardiomyocytes in various clinical settings (e.g. myocardial infarction, AHF, stroke etc.) and is influenced by numerous clinical factors (e.g. renal function, coronary artery disease, ethnicity etc.) different levels of troponin are found in each of these clinical scenarios [9, 25, 26]. As a consequence, not one specific cut-off can uniformly be applied as diagnostic or prognostic measure in all these settings. The high percentage of patients showing hs-TnT values above the conventional hs-TnT limit (99^th^ percentile URL of 14 ng/l) in the present analysis demonstrates that such cut-off is of limited value for any risk prediction in this subset of AHF patients. The specificity of the 99^th^ percentile URL for 30-day mortality was 13% in HFpEF patients and even lower in those with HFmrEF and HFrEF indicating that higher values need to be used for an adequate prognostication. This is also underlined by previous AHF studies. Parissis et al. identified a ROC optimized hsTnT cut-off of 77 ng/l to predict all-cause mortality with a sensitivity of 62% and specificity of 72% in patients with predominantly reduced LV-EF [27]. Roset et al. found a ROC optimized hsTnT cut-off of 35 ng/l with a sensitivity of 67% and specificity of 56% in a population consisting of both HFrEF and HFpEF [28]. The difference in cut-offs among these studies may be due to the assessment of different patient cohorts. The lower hsTnT limit in the analysis from Roset et al. is possibly based on the inclusion of AHF patients showing also HFpEF as well as less comorbidities Fig. 4.

However, no data are available up to now regarding a potential difference in the accuracy of hs-TnT based risk prediction in different HF groups (HFpEF vs. HFmrEF vs. HFrEF) of AHF patients. Sanders-van Wijk et al. assessed the association of hs-TnT with 18-months survival both in HFpEF and HFrEF patients. In the HFpEF group, an OR with very wide 95%-CI and a p-value of 0.02 was found. In contrast, in the HFrEF group a highly statistical significance was observed (OR 2.53, 95%-CI 1.76–3.64; *p* < 0.001). A direct comparison was not conducted. The present results from ROC analysis show the optimal cut-off of hs-TnT for clinical outcome prediction in AHF to be markedly higher in HFmrEF and HFrEF patients compared to HFpEF patients. In the light of this finding, troponin elevations should be interpreted differently depending on the presence of HFpEF compared to HFmrEF/HFrEF. The optimal cut-off of hs-TnT for 30-day mortality was two-fold higher in HFmrEF and HFrEF (60 ng/l and 54 ng/l respectively) than in HFpEF (29 ng/l).

Importantly, the accuracy of hs-TnT based prognostication was significantly higher in the HFmrEF and HFrEF group compared to the HFpEF group. The limited prognostic potential in HFpEF patients was mainly caused by a relatively low specificity of 52%. In addition, statistical significance was not reached in the adjusted regression analysis for HFpEF patients assessing the independent association of hs-TnT with 30-day mortality. This is possibly explained by the marked heterogeneity in the pathophysiological causes of HFpEF including hemodynamic, structural, metabolic and inflammatory alterations [29]. In such a variety of disorders one marker alone may not be sufficient to adequately predict clinical outcome.

### Limitations

The work represents a single-center analysis and, hence, generalization of the results should be performed with caution. Due to the retrospective design unknown residual confounders cannot be ruled out. Although the number of patients in the overall study was large stratification into three HF categories created groups of rather limited size relative to the number of events. Since NT-pro-BNP was not routinely measured in all patients, combination with this parameter to enhance prognostication was not possible. No other assays of cardiac troponins were used and, therefore, only hs-TnT were analyzed for outcome prediction. Long-term outcome could not be assessed due to the lacking follow-up beyond 30 days.

## Conclusion

The present study demonstrates that abnormal hs-TnT levels a very common in AHF patients and higher cut-offs need to be used for adequate 30-day outcome prediction than the 99th percentile URL. This is particularly true for HFmrEF and HFrEF patients in whom the optimal hs-TnT cut-off was found to be two-fold higher than in HFpEF patients. Most importantly, hs-TnT shows significantly better predictive performance in HFmrEF and HFrEF compared to HFpEF in patients with AHF.

## Supplementary Information

Below is the link to the electronic supplementary material.

Supplementary file1 (DOCX 13 KB)
