# Supplementary material for: Prognostic impact of high-sensitive troponin on 30-day mortality in patients with acute heart failure and different classes of left ventricular ejection fraction
Source: Heart Vessels. 2022 Jan 15;37(7):1195–202. doi: 10.1007/s00380-022-02026-x (PMC9142424; doi:10.1007/s00380-022-02026-x)
Supplement: Supplementary file 1 — Supplementary file1 (DOCX 13 KB) [file 380_2022_2026_MOESM1_ESM.docx]

Supplemental Table S1: Multivariable linear regression for the identification of factors independently associated with hs-TnT

|  | HFpEF | | HFmrEF | | HFrEF | |
| --- | --- | --- | --- | --- | --- | --- |
|  | Regression coefficient ß | p-value | Regression coefficient ß | p-value | Regression coefficient ß | p-value |
| Sex | - | - | - | - | 0.028 | 0.04 |
| Diabetes mellitus | 0.008 | 0.25 | - | - | - | - |
| Atrial fibrillation | -0.021 | <0.01 | -0.035 | 0.07 | -0.033 | 0.01 |
| Coronary artery disease | 0.017 | <0.01 | - | - | - | - |
| Previous PCI | - | - | 0.027 | 0.13 | - | - |
| Previous MI | 0.012 | 0.26 | - | - | - | - |
| Peripheral edema | - | - | -0.024 | 0.23 | - | - |
| eGFR | -0.001 | <0.001 | -0.001 | 0.01 | -0.001 | <0.01 |

eGFR: Estimated glomerular filtration rate; HFpEF: Heart failure with preserved ejection fraction; HFmrEF: Heart failure with mid-range reduced ejection fraction; HFrEF: Heart failure with reduced ejection fraction; Hs-TnT: High-sensitive troponin T; MI: Myocardial infarction; PCI: Percutaneous coronary intervention
